# Introducing Routine Assessment of Adverse Childhood Experiences For Looked-After Children: The Use and Properties of the Trauma and Adverse Life Events (TALE) Screening Tool

**DOI:** 10.1007/s40653-023-00559-5

**Published:** 2023-06-28

**Authors:** Asa Kerr-Davis, Saul Hillman, Katharine Anderson, Richard Cross

**Affiliations:** 1 Assessment and Therapy, Five Rivers Child Care Limited, Salisbury, Wiltshire UK; 2 Research Department of Clinical, Educational and Health Psychology, University College London, London, UK; 3 Child Attachment and Psychological Therapies Research (ChAPTRe), Anna Freud Centre, London, UK

**Keywords:** Adverse childhood experiences, ACEs measure, Looked-after children, Trauma

## Abstract

The present study aims to illustrate the process of developing, implementing, and clinically validating a new assessment measure, the Trauma and Adverse Life Events (TALE) screening tool, to assess Adverse Childhood Experiences (ACEs) among looked-after children. The TALE was developed by adapting existing ACEs measures to reflect the experiences of looked-after children. The TALE was completed by the local authority social worker for 218 children placed with Five Rivers Child Care (a UK fostering agency, residential, and educational care provider). Reliability was examined and exploratory factor analysis was conducted. Correlations between TALE scores, background variables, and psychosocial wellbeing using the carer-report Strengths and Difficulties Questionnaire (SDQ) and Child Dissociative Checklist (CDC) were also explored. The TALE was found to have acceptable reliability (*α* = .71). A three-factor solution was found which explained 46.24% of the variance, with factors labelled ‘Direct Experience of Abuse’, ‘Witnessing Harm’, and ‘Household Dysfunction’. Exposure score was significantly associated with total difficulties score on the SDQ *(r*_*s*_ = .24, *p* < .001) and Impact score was associated with the SDQ’s impact score (*r*_*s*_ = .33, *p* < .001). Exposure and Impact scores were both positively correlated with CDC scores (*r*_*s*_ = .16, *p* = .021 and *r*_*s*_ = .22, *p* = .002). This paper presents evidence of the importance of screening looked-after children for ACEs and demonstrates that the TALE is a valid and reliable tool for this purpose. Adverse and traumatic experiences were highly prevalent in this population and appeared to be closely related with children’s psychosocial wellbeing. Results emphasise the importance of routine assessment of past experiences within trauma-informed psychological care and intervention planning for looked-after children.

## Introduction

### Adverse Childhood Experiences and Outcomes

The last twenty years have seen a surge in research about the negative sequelae of Adverse Childhood Experiences (ACEs) across the lifespan. The seminal ACEs study by Felitti et al. (1998) demonstrated an association between adversity in childhood and a range of negative health outcomes in a sample of over 17,000 American adults. Childhood experiences explored included different types of abuse, neglect, and household dysfunction (e.g., living with a mentally unwell adult; domestic violence; substance misuse; parental incarceration; and parental separation). A dose–response effect was observed: the greater the number of ACEs an adult had experienced, the greater their risk of negative health outcomes in adulthood (Bethell et al., 2017). As a result, ACEs assessments have come to be widely used to generate a cumulative measure of adversity-related risk for adult health outcomes (Shonkoff & Garner, 2012).

Although the first ACE studies focused only on physical health outcomes, subsequent replications in England (Hughes et al., 2017), Wales (Bellis et al., 2015a, b), and Scotland (Smith et al., 2016) have consistently demonstrated that experience of four or more ACEs is strongly associated with negative outcomes both for physical health and psychosocial problems, such as sexual risk taking, mental ill health, problematic substance use; interpersonal violence; and self-harm (Björkenstam et al., 2017; Hughes et al., 2016, 2017). The ACEs framework proposes that stressful childhood experiences can disrupt the neurodevelopment of the child by chronic elevation of stress hormone levels, leading to dysregulation of developing neurobiological systems (Bucci et al., 2016; Cohen et al., 2002). In turn, a dysregulated stress response leads to a heightened propensity for social, emotional, and cognitive problems alongside challenging and risky behaviour (D’Andrea et al., 2012). Through this mechanism, chronic trauma exposure in childhood is associated with psychological difficulties including posttraumatic stress, anxiety, and depression symptoms (Briggs-Gowan et al., 2010). Childhood adversity and adult mental health problems have been robustly associated on an international scale – a large analysis by Kessler et al. (2010) of data from 51,945 adults derived from the World Mental Health Surveys in 21 countries found that at least one third of diagnosed mental health conditions were directly related to ACEs (Green et al., 2010).

A growing body of research has demonstrated that the deleterious psychosocial effects of ACEs can be observed long before adulthood (Oh et al., 2018). Among adolescents, experience of sexual abuse by attachment figures is robustly associated with functional impairments with interpersonal relationships (Daignault & Hébert, 2009), and a more recent study of over 3,000 15-year-olds found that each additional traumatic event experienced during childhood increases the likelihood of high-risk behaviour by 6–22% (Layne et al., 2014). This research, replicating the dose–response effect detected by Felitti et al., endorses the notion that ACEs have a cumulative impact on functioning, even before adulthood. There is a lack of evidence that explores whether there is a causal relationship between ACEs and mental health difficulties, but persistent abuse and neglect during sensitive periods of development have been associated with disturbance in early attachments and disruption to children’s long-term development, yielding negative psychological outcomes (Tarren-Sweeney, 2008).

### Looked-After Children, Trauma and Adversity

As of March 2020, there were 80,080 looked-after children in the United Kingdom, 65% of whom entered care due to experiencing abuse or neglect (UK Government, 2020). Maltreatment, including neglect and emotional, physical, and sexual abuse, represent distressing sources of adversity for children which can cause lasting psychological trauma (Briggs et al., 2012; Bush, 2018). The high prevalence of traumatic experiences among looked-after children is well-documented, with reported rates of past exposure to at least one form of trauma exceeding 90% for young people in foster care (Dorsey et al., 2012; Stein et al., 2001). Traumatic experiences also vary by gender, with males in foster care more likely to have experienced interpersonal violence and females more likely to experience sexual trauma (Salazar et al., 2013). As Simkiss (2019) notes, children largely enter care *because* of ACEs and looked-after children have often experienced the ‘toxic trio’: parental domestic violence, substance misuse, and mental illness (Rahill & Hendry, 2014). Moreover, children removed from their birth parents after infancy intrinsically lack the opportunity to develop a secure attachment from a consistent caregiver, which is a well-evidenced protective factor in the face of childhood trauma (Cheong et al., 2017; Liebermann et al., 2005).

It is perhaps unsurprising, therefore, that research has consistently demonstrated that this population, with a high level of adversity and trauma, exhibits a high level of psychological difficulties (Rebbe et al., 2017; Simkiss, 2012). Estimates of the exact proportion of looked-after children who meet diagnostic criteria for psychiatric disorders vary, but there is consensus that the figure is disproportionately high for this population – between 48 and 69% (Luke et al., 2014; Sawyer et al., 2007). A report commissioned by the National Society for the Prevention of Cruelty to Children (NSPCC) found that looked-after children are 4–5 times more likely to have a diagnosable mental health condition than their peers (Bazalgette et al., 2015; NICE, 2010), and a national survey of looked-after children’s mental health in the UK identified conduct disorder as an especially prevalent condition (Meltzer et al., 2003). Given the high rates of exposure to environmental adversity during early childhood for looked-after children, rates of post-traumatic stress disorder (PTSD) may also be elevated (Chambers et al., 2010; Anthony et al., 2022). Indeed, Morris et al. (2015) found PTSD-like symptoms in 75% of their sample of looked-after children. Although this study had a small sample of n = 27 and used a brief screening tool rather than robust psychiatric diagnosis, it supports findings from a large study by Ford et al. (2007) of 1453 children, which found looked-after young people were 19 times more likely to have a PTSD diagnosis than peers. Despite this strong evidence of elevated psychological problems among looked-after children, further research is required to explore the reasons for discrepancies in prevalence findings between studies.

One reason for the relative diversity of estimated prevalence rates, besides the variable quality of sampling and diagnostic techniques, could be that the mental health difficulties of looked-after children are not completely congruent with criteria for traditional psychiatric diagnosis (DeJong, 2010). Growing evidence suggests that trauma and adversity during sensitive developmental periods can lead to a cascade of attachment- and trauma-related symptomatology, such as persistent emotional dysregulation, disorganised attachments, and difficulties with attention and conduct. One robust cluster analysis by Tarren-Sweeney (2013) demonstrated that 35% of his sample in foster care had difficulties which fit into traditional psychiatric categories, but a further 20% displayed complex attachment- and trauma-related symptoms which did not fit the conceptual categories of the Diagnostic Statistical Manual (DSM 5 – see American Psychiatric Association, 2013).

This analysis demonstrates that the complex psychosocial impacts of trauma on child development may be better captured by emerging diagnostic concepts, not yet accepted by the DSM, such as Developmental Trauma Disorder or ‘complex trauma’, rather than the traditional categories of PTSD and Reactive Attachment Disorder (Bremness & Polzin, 2014; Herman, 2015; John et al., 2019; van der Kolk, 2005). Ai et al. (2013) have argued that fully understanding the impact of ACEs on the development of children in care requires appropriate categories for the characteristic behavioural, social, cognitive, and emotional difficulties experienced by these young people. As one example, Dann (2011) argues that conceptualising the symptoms of ‘conduct disorder’ within the context of previous trauma would both reduce barriers to accessing services and help teachers and other professionals to understand children’s behavioural presentation. In essence, to understand a child’s presentation it is first necessary to understand their history of adversity and trauma (Cross, 2011).

### Screening Looked-After Children for Trauma and Adverse Life Events

Assessments for looked-after children have received increased scrutiny in recent years, and statutory guidance in the UK now reflects the view that looked-after children should receive mental health assessments as a priority (Department for Education, 2015; Rahilly & Hendry, 2014). It is now generally accepted that the key adults in the day-to-day care of looked-after children – foster carers, residential workers, and social workers – must have a clear understanding of the child’s history (including ACEs) to understand relevant underlying factors which may influence the child’s presentation (Cross, 2012; Larkin et al., 2014; Murphey & Bartlett, 2019). The trauma-informed care approach, which moves beyond a diagnostic model of care to focus on developing a physically and psychologically safe environment, emphasises assessment of each child’s traumatic history as an essential component of a wider routine mental wellbeing assessment process (Bloom, 2013; Ko et al., 2008; Lang et al., 2017).

Several instruments have been developed which screen for PTSD *symptoms* in response to acute trauma exposure (Perfect et al., 2016). In a robust systematic review of the recent literature, Eklund et al. (2018) identified and reviewed 18 measures designed to screen for trauma in children, but none were designed specifically for the care population, and several measures *only* address PTSD symptomatology rather than trauma exposure or developmental trauma symptoms. Eklund et al. concluded that there is very little psychometric evidence to support using symptom-based trauma screening measures for school-aged children. One specialist assessment tool, the Assessment Checklist for Children (Tarren-Sweeney, 2007, 2014), has been developed to assess the characteristic psychosocial difficulties which may be experienced by children in care. This 120-item tool measures trauma exposure, attachment-related behaviour, emotional states, and traits relating to others. Whilst this measure provides a valuable assessment method for symptomatic difficulties, it could be considered impractical due to its length. Therefore, a condensed tool is needed which captures looked-after children’s experience of trauma and adversity.

### Development of the TALE

The Trauma and Adverse Life Events screening tool (TALE) was developed in collaboration between Five Rivers Child Care Ltd (FRCC) and the Anna Freud National Centre for Children and Families (AFNCCF) in the UK. As a fostering agency and residential care provider, FRCC has implemented a comprehensive Attachment- and Trauma-Informed Care model (ATIC®), which includes annual emotional and behavioural wellbeing assessments for every child. The battery of assessments includes the Strengths and Difficulties Questionnaire (SDQ – Goodman, 2001; Goodman & Goodman, 2012), the Attachment Screening Assessment (ASA – Glaser et al., 2011), the Relationship Problems Questionnaire (RPQ – Minnis et al., 2002), the Child Dissociation Checklist (CDC – Putnam & Peterson, 1994), and the Health of the Nation Outcomes Scale Child and Adolescents (HoNOSCA – Pirkis et al., 2005). The information gleaned from these screening assessments is summarised and reported to clinicians (psychologists and psychotherapists), who use it to inform the clinical formulation and conversations in an Integrated Case Management Meeting with the carer, supervising social worker, education representative, and the local authority social worker. Whilst developing this process, it was deemed important to include a measure of ACEs as a brief screening tool to inform the clinician’s understanding of each child’s trauma and adversity history (McLennan et al., 2020).

FRCC sought to identify suitable ACEs measures, but an informal review found that no existing measure was adequate or appropriate for use with the looked-after children population. The comprehensive review of ACEs measures by Bethell et al. (2017) illustrates that most ACE measures rely on adult self-report of historic experiences, and those which explore children’s experiences tend to use parent-report which is not possible for looked-after children (e.g., National Survey of Children’s Health ACEs, Bethell et al., 2014). As carers may only receive a partial chronology of a child’s known life experiences, a measure was required which local authority social workers could complete. The child’s social worker is likely to have the most detailed information about the child’s early life before entering care, and, according to best-practice guidelines, comprehensive assessment processes should draw on multiple informants with different viewpoints of the child (Luke et al., 2014).

No ACEs measures could be found which i) were specific to the experiences of the looked-after children population; ii) were designed for completion by a social worker rather than a parent; iii) excluded items which are less relevant to the looked-after children population; iv) included an indicator of the impact of ACEs on current behavioural presentation; and v) were designed for use as routine screening tools within a wider psychological assessment process rather than research instruments. It was therefore necessary to develop a measure which was more appropriate and reflective of the experiences of looked-after children.

To measure exposure to ACEs, the first part of the TALE (the Exposure section) was developed by adapting the original ACEs tool used by Felitti et al. (1998), reducing the total number of items to increase convenience and maximise the return rate. The FRCC Assessment & Therapy team rephrased some items based on clinical experience to better reflect the experiences of looked-after children, but these minor changes sought to avoid compromising the psychometric properties of the original measure. For brevity, questions from generic ACEs screening tools about traumatic stressors which are less relevant to the UK looked-after children population, such as exposure to natural disasters and war, were omitted (e.g., Sachser et al., 2017).

There has been some recent criticism of the use of ACE scores as ‘crude measures’ of childhood stress exposure which cannot meaningfully calculate risk for an individual (Anda et al., 2020). This criticism proposes that the *impact*, *intensity,* and *chronicity* of exposure is important, not simply the total ACEs score (Bartlett, 2020). In response to this perspective, the TALE includes an innovate Impact section, which allows the respondent to rate the severity of the impact which each itemised experience is thought to have had on the child. The Exposure and Impact scores are reported to the FRCC psychologist at the granular level to inform their understanding of the impact which traumatic and adverse events may have had on the child and to inform therapeutic care planning (Fratto, 2016).

### The Present Study

This study draws on routine assessment data collected by FRCC, an independent fostering agency, education, and residential care provider, to explore the internal and external psychometric properties of the TALE.

The characteristics of the sample were investigated, including the average number of ACEs which the TALE identified. The internal properties of the TALE Exposure section were then investigated, including its internal reliability and the factor structure. The structure of the Impact section was not investigated because it is not intended to contain a latent structure: rather, its purpose is to provide additional detail about the extent to which each experience has impacted a child’s wellbeing. Relationships between TALE scores and background variables, including gender, age at admission to care, and placement alongside a sibling, were examined to explore how a high degree of adversity in childhood may affect a child’s journey into care. Additional associations between number of adverse childhood experiences and psychosocial difficulties, including dissociative behaviours and emotional and behavioural wellbeing, were investigated to provide an indication of the TALE’s predictive validity.

## Research Questions

1. What are the internal properties and factorial structure of the Exposure section of the TALE?
2. Are higher Exposure and Impact scores on the TALE related to background and demographic variables including age at entry to care, gender, and placement alongside a sibling?
3. Are children with higher Exposure and Impact scores on the TALE more likely to experience:
  (i) Psychosocial difficulties measured using the carer-report SDQ?
  (ii) Dissociative difficulties related to a trauma response measured using the carer-report CDC?

## Methodology

### Design

This quantitative cross-sectional study used routine assessment data collected by FRCC to examine the development, internal structure, and external psychometric properties of the TALE. This study is nested within a larger evaluation project exploring assessment and outcomes of children who are looked-after within FRCC.

### Participants

Inclusion criteria for this study were broad, and all children who were placed with FRCC fostering or residential services were considered eligible if their social worker had completed a TALE assessment for them. Children were excluded if: (i) their carers or social workers did not consent to their participation (*n* = 0); (ii) their social worker returned the TALE only partially completed (*n* = 7).

Local authority social workers returned the TALE for 218 children. Participants were aged between 0 and 17 years (*M* = 9.40, *SD* = 3.52), of whom 18.6% were aged 0–5; 47.7% were aged 6–10; and 33.6% were aged 11–18.

**Fig. 1:**
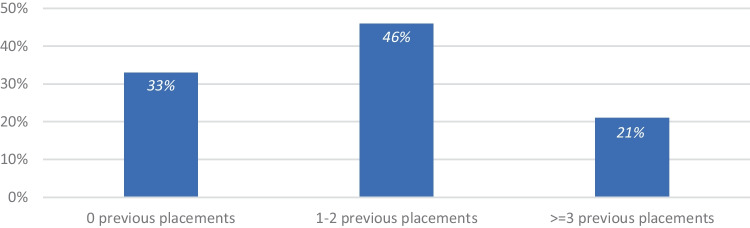
Participants by number of placement transitions (*n* = 191, missing = 27)

123 (56%) were male and 95 (44%) were female. Participants were predominantly White (90%), and 10% were from Black, Asian, or other minoritized ethnic (BAME) backgrounds. 23 were living in residential care and 195 were in foster care placements. 120 were placed with siblings and 94 were classed as ‘singletons’ (missing *n* = 4). Figure 1 shows the number of times participants had moved placements – the maximum number was 9.

Participants were receiving a range of support for their mental health and educational needs. Excluding missing data (*n* = 51), 66 of 167 children (40%) had an Education Health Care Plan, and 92 of 165 children (56%) were receiving medication or therapeutic support from NHS mental health services or in-house therapy from FRCC clinicians.

### Measures

The TALE (Hillman et al., 2018) screening measure is a brief social worker-report tool used to explore the extent of adversity and trauma which a child in care may have experienced in their childhood. The TALE comprises two sections: an Exposure part and an Impact part.

The Exposure section measures the extent of trauma and adversity experienced, with a reduced set of items adapted and rephrased from the original Adverse Childhood Experiences tool used by Felitti et al. (1998). The constructs measured include experience of neglect, abuse, and household dysfunction (including parental substance misuse, mental illness, domestic violence, separation, and incarceration). There are 1–2 questions per construct and 14 questions in total. For each item, the respondent can select ‘Not known’, ‘Possible’, or ‘Definite’. There is no option for ‘Definitely did not experience’, because one cannot be certain of what a child in care has *not* experienced. Two scores are derived: a definite score, which equals one point for each tick in the ‘Definite’ column; and a potential score, which combines the number of ticks in the ‘Definite’ and ‘Possible’ column.

The second part of the TALE is an Impact section, which allows the respondent to rate the severity of the impact which each item is thought to have had on the child – whether ‘Mild’, ‘Moderate’, or ‘Severe’. To provide an indicator of the overall impact of trauma and adversity on each child, a composite Impact score is computed by summing the number of experiences rated as having a mild (1 point), moderate (2 points), or severe impact (3 points), producing a maximum possible score of 42 across the 14 items. The present study aims to establish the preliminary psychometric properties of the TALE.

The SDQ is a carer-report screening measure which assesses psychosocial problems in children aged 2–17 (Goodman, 2001). The 25 items form five subscales: (i) emotional difficulties, (ii) behavioural/conduct problems, (iii) hyperactivity/ inattention, (iv) peer problems and (v) prosocial behaviour, the first four of which combine to produce a total difficulties score. The threshold score for clinical significance is 17. The SDQ has good concurrent and discriminant validity (Goodman & Scott, 1999; Muris et al., 2003) and good test–retest reliability (Stone et al., 2015). Due to the poor internal consistency of some subscales (*α* < 0.7), it is recommended that only the total difficulties score is used for screening purposes with younger children, but the subscales are reliable for older children (Mieloo et al., 2012). In the present study, the SDQ subscales were included in the exploratory analysis to examine relationships between past experiences and different types of internalising and externalising presentations.

The CDC (Putnam, 1993; Putnam et al., 1993) is a carer-report measure of children’s behaviours which may indicate psychological dissociation. The CDC contains 20 items which are rated on a Likert scale of 0–2 (‘Not True’, ‘Sometimes/ Somewhat True’, ‘Very True’) based on the child’s behaviour in the last 12 months. Although this measure is a screening tool rather than a diagnostic assessment, a clinical cut-off score of 12 has been proposed (Wherry et al., 1994). The CDC has acceptable test–retest reliability (Putnam et al., 1993), strong internal consistency, good convergent and discriminant validity (Putnam & Peterson, 1994), and good concurrent validity (Wherry et al., 1994).

### Procedure

In line with the FRCC routine baseline assessment process, the TALE was sent to the local authority social worker for each child after being placed with FRCC for 6 weeks. The SDQ and CDC were sent separately to the foster carer or residential key worker, and completed forms were requested for return via email within two weeks. If the measures were not returned within one month, researchers at FRCC followed up with a prompting email. For some children, the TALE was returned without other measures, so the value of *n* varies in the results presented below. Data from the measures were inputted in SPSS as an anonymised and encrypted dataset. Background information gleaned from each child’s referral documentation was extracted from the FRCC internal database and added to the assessments database. This file was shared with the Senior Research Consultant at AFNCCF who supported the analysis and write-up of this study.

### Ethical Considerations

This study forms part of a wider study commissioned by FRCC which was granted ethical approval by University College London (UCL). All the data included in this study was collected as part of the FRCC routine assessment process, which monitors outcomes and facilitates interventions for children in FRCC’s care. Due to the age of the participants, local authority social workers provided informed consent for children’s anonymised data to be used for research purposes, and foster carers completed additional routine assessment measures and returned them via email to a designated secure inbox which only the authors have access to. All data were anonymised and inputted into a database which was shared via encrypted email with the Senior Research Consultant at AFNCFF.

### Statistical Analysis

SPSS version 28 was used to conduct the analysis. An initial summary of descriptive statistics was followed by an exploratory factor analysis of the Exposure section of the TALE to explore its underlying structure. A Shapiro–Wilk test showed that the distribution of Exposure scores followed a non-normal distribution (*W* = .972, *p* < .001). As a result, non-parametric statistical tests including the Mann–Whitney U test and Spearman’s rank correlation coefficient were used to explore the relationship between Exposure scores, Impact scores, and demographic, psychosocial, and background variables.

## Results

### Descriptive Statistics

Social workers returned TALE assessments for a total sample of 218 children, as required by the FRCC baseline assessment process. Of these, 206 (94.5%) had ‘potential’ Exposure scores of 5 or more (the sum of ‘definite’ and ‘possible’ scores), whereas 12 had ‘potential’ Exposure scores of 4 or fewer (5.5%). On average, social workers reported 4.84 experiences which had ‘definitely’ happened (*SD* = 2.49) and 5.22 which were ‘not known’ whether they had happened or not (*SD* = 2.70). The mean Impact score was 23.62 (*SD* = 7.42) out of a possible 42. Henceforth, the term ‘Exposure score’ refers to the ‘definite’ Exposure score only.

### Internal Reliability and Factor Structure of Exposure Scale

Overall reliability of the Exposure section was *α* = .71, based on *n* = 196 with 22 cases removed by listwise deletion due to missing data. This internal consistency surpasses the conventionally accepted threshold of .70 (DeVellis, 2003). 196 cases were included in an exploratory factor analysis of the Exposure section of the TALE, equating to a ratio of more than 10 cases per variable. The data satisfied the following criteria to assess factorability (Costello & Osborne, 2005):

(i) All items correlated at least .3 with at least one other item.
(ii) Diagonals of the anti-image correlation matrix all exceeded .5.
(iii) Bartlett’s test of sphericity was significant (*χ*^*2*^ = 602.08, *p* < .001).
(iv) The Kaiser–Meyer–Olkin Measure of Sampling Adequacy exceeded the conventionally accepted value of 0.6 (*KMO* = .67).

A principal components analysis (PCA) indicated a possible five components with eigenvalues greater than 1.00. However, a parallel analysis subsequently suggested that only three of these components had eigenvalues greater than those randomly generated. A scree test further indicated that a three-factor solution was preferable. The initial PCA was repeated with factors fixed at 3 and direct oblimin rotation, to determine whether the matrix was orthogonal. The resulting component correlation matrix showed that only one factor was greater than .50, indicating that the matrix was orthogonal. Thus, a final PCA was run using varimax rotation which yielded a three-factor solution which accounted for 46.24% of the variance.

The components of this structure each had face validity, and items could be meaningfully grouped and labelled. The first factor contains 7 items which describe the experience of direct abuse, including physical, verbal, emotional, and sexually abusive experience. For example, ‘*Has the child been physically hurt in some way by a parent or adult?*’; ‘*Has the child been exposed to a parent or other adult being verbally abusive to them?*’. This factor had an eigenvalue of 3.28 and accounted for 23.41% of the variance, with factor loadings ranging from .32 to .78. It was labelled ‘Direct Experience of Abuse’.

The second factor was composed of 5 items which were conceptually linked by the notion of witnessing others experiencing harm or separation. For example, ‘*Has a close family member of the child either been killed or seriously hurt?*’; ‘*Has the child ‘lost’ a close family member through divorce, abandonment, or another reason*?’. This 5-item factor had an eigenvalue of 1.77, accounting for 12.66% of the variance with factor loadings ranging from .31 to .82 and was labelled ‘Witnessing Harm’.

The final factor included 3 items which were closely connected as indicators of household dysfunction, such as ‘*Has the child been looked after by a parent who was often under the influence of alcohol or other drugs?*’; ‘*Has the child been looked after by a parent that was mentally unwell?*’. This factor had an eigenvalue of 1.42 and accounted for 10.17% of the variance, with factor loadings ranging from .49 to .83. This factor was labelled ‘Household Dysfunction’.

Despite the cogence of this factorial solution, only the first factor had acceptable reliability (*α* = .76). Factors two and three had *α* = .57 and *α* = .58 respectively (see Appendix). Although the deletion of item 13 (‘*Has a close family member of the child been to prison?*’) from factor three increased reliability to .65, this remained beneath the acceptable threshold of .70. None of the correlations between factors exceeded .50. The only item which was not included within any factors was item 2 (‘*Has the child ever suffered from a serious or life-threatening illness which has had an impact on their life?*’). Due to the exploratory stage of this measure’s development, both the three-factor solution and the one-factor solution using the overall Exposure score were explored in further analyses.

### Relationship to Background Variables

Boys and girls had very similar mean Exposure scores (*M* = 4.79, *SD* = 2.74 and *M* = 4.90*, SD* = 2.12 respectively), and the difference between genders was found not to be statistically significant (*U* = 5532.50*, p* = .50). Bivariate analysis indicated that there was no significant correlation between age at admission to care and Exposure score (*r*_*s*_ = -.008, *p* = .91) or Impact score (*r*_*s*_ = -.08, *p* = .235). However, age at admission to care was significantly positively correlated with ‘Direct Experience of Abuse’ (*r*_*s*_ = .16, *p* = .027), and negatively correlated with ‘Household Dysfunction’ (*r*_*s*_ = -.14, *p* = .047).

Exposure score was found to be significantly associated with number of previous placements, with a weak effect size (*n* = 188, *r*_*s*_ = .144, *p* = .049). Of the Exposure factors, only factor two (‘Witnessing Harm’) was weakly correlated with number of previous placements (*r*_*s*_ = .15, *p* = .044). Moreover, Impact score was significantly positively correlated with number of previous placements (*r*_*s*_ = .19, *p* = .009).

On average, children placed in a home alongside a sibling (*n* = 94) had Exposure scores of less than 5 (*M* = 4.32, *SD* = 2.05), whereas children placed alone (*n* = 120) had experienced more than 5 ACEs (*M* = 5.13, *SD* = 2.66). This discrepancy was found to be statistically significant (*U* = 4575.50, *p* = .017). Moreover, children placed alone had significantly higher average Impact scores than children placed with a sibling (*U* = 4246.50, *p* = .009).

### Relationship to Psychosocial Difficulties

An SDQ was completed by the foster carers of 210 of the children in the present sample, 44.8% of whom (*n* = 99) scored on or above the clinical cut-off of 17, indicating that clinically significant behavioural and emotional problems were present. A significant positive correlation was found between TALE Exposure score and total difficulties score on the SDQ, with a small effect size *(r*_*s*_ = .24, *p* < .001). Additionally, TALE Exposure score was positively associated with the likelihood of the SDQ predicting a diagnosis (*r*_*s*_ = .25, *p* < .001), and the Impact score on the TALE was also positively associated with the SDQ’s impact score (*r*_*s*_ = .33, *p* < .001) and prediction of diagnosis (*r*_*s*_ = .26, *p* < .001). Although the TALE factor ‘Direct Experience of Abuse’ was significantly positively correlated with total SDQ score (*r*_*s*_ = .23, *p* = .001), neither ‘Witnessing Harm’ nor ‘Household Dysfunction’ were significantly associated with total SDQ score (*r*_*s*_ = .09, *p* = .192 and *r*_*s*_ = .09, *p* = .186 respectively). See Table 1 for a matrix of the correlations between all TALE constructs and SDQ subscales and impact scores.

**Table 1:** Correlations between TALE constructs and SDQ subscales

		**TALE Overall**	**Overall Impact**	**‘Direct Experience of Abuse’**	**‘Witnessing Harm’**	**‘Household Dysfunction’**
**SDQ Total**	*r*_*s*_	.239**	.234**	.228**	.094	.093
	*p*	**< .001**	**< .001**	**.001**	.192	.186
**SDQ Impact Score**	*r*_*s*_	.242**	.327**	.287**	.164*	.106
	*p*	**< .001**	**< .001**	**< .001**	**.023**	.136
**SDQ Emotional Problems**	*r*_*s*_	.127	.149*	.177*	.004	.088
	*p*	.066	**.033**	**.013**	.959	.213
**SDQ Conduct Problems**	*r*_*s*_	.210**	.203**	.137	.142*	144*
	*p*	**.002**	**.004**	.057	**.048**	**.042**
**SDQ Hyperactivity/ Inattention Problems**	*r*_*s*_	.160*	.198**	.145*	.087	.071
	*p*	**.020**	**.004**	**.043**	.227	.314
**SDQ Peer Relationships Problems**	*r*_*s*_	.170*	.132	.215**	.027	-.012
	*p*	**.014**	.060	**.003**	.709	.860
**SDQ Prediction of Any Diagnosis**	*r*_*s*_	.252**	.258**	.217**	.139	.112
	*p*	**< .001**	**< .001**	**.003**	.054	.116

^*^p<.05; ^**^p<.01

The CDC was completed by foster carers for 205 of the children in the sample, of whom 136 (61.5%) scored above the clinical threshold indicative of dissociative difficulties (score of 12 or more). TALE Exposure score was significantly positively correlated with CDC score (*r*_*s*_ = .16, *p* = .021) and the ‘Direct Abuse’ factor (*r*_*s*_ = .22, *p* = .002), but not the ‘Witnessing Harm’ (*r*_*s*_ = .24, *p* = .237) or ‘Household Dysfunction’ factors (*r*_*s*_ = .13, *p* = .061). Impact score on the TALE was also significantly correlated with total CDC score (*r*_*s*_ = .22, *p* = .002).

## Discussion

### Measuring ACEs for Looked-After Children

The need to measure and account for Adverse Childhood Experiences (ACEs) in routine mental health assessments has become a pressing, yet contentious, issue in recent years (Danese, 2019). Whilst correlational evidence indicates a clear link between childhood adversity and subsequent physical and mental health difficulties in adulthood (Felitti et al., 1998), the use of ACEs screening measures has remained divisive, with some critics suggesting that such measures fail to capture the impact of chronicity or severity of childhood adversity or to predict specific later life outcomes (Anda et al., 2020). The present study does not seek to demonstrate the utility of ACEs measures as a universal feature of health screening. On the contrary, the purpose of the present study was to illustrate the development and validation of a measure, based on the ACEs framework, which provides specific, clinically relevant insight into the past experiences of looked-after children. The purpose of screening for adversity in this context is not to predict future health outcomes, but to provide valuable information about the context of looked-after children’s current presenting behaviours to support psychologists and psychotherapists during the case formulation and intervention planning process (Cross, 2012). This provides a parsimonious, pragmatic, and clinically useful alternative to accessing case notes in order to understand each child’s past experiences.

### Psychometric Properties of the TALE

The TALE was developed as a unique social worker-report measure of both the extent of exposure to adverse events and the impact of these experiences on looked-after children. Items were adapted from existing ACEs measures, maintaining overall construct validity, and the tool was designed to reflect the specific experiences of the looked-after population. In line with previous research, the level of exposure to adverse and traumatic events was high (Simkiss, 2019) – 94.5% of the sample had either ‘possibly’ or ‘definitely’ experienced more than five. It is worth noting that, on average, social workers reported that they ‘did not know’ whether or not the child had experienced several of the items (*M* = 5.22, *SD* = 2.70). This illustrates the often-opaque nature of looked-after children’s histories, and the importance of gathering information from the best-informed professionals in the child’s life. The analyses reported in this paper only used the ‘definite’ TALE score, but it is very likely that this is an underestimate of the real extent of trauma and adversity exposure in this population.

Overall, the TALE Exposure scale was demonstrated to have internal consistency which surpassed the acceptable threshold of *α* = .70 (DeVellis, 2003) and exploratory factor analysis revealed an underlying structure comprising three factors which accounted for 46.24% of the variance. These three factors indicate that the adverse experiences on the TALE can be meaningfully grouped into types of abuse which directly target and harm the child (‘Direct Experience of Abuse’); an environment in which the child witnesses the suffering of, or separation from, others (‘Witnessing Harm’), and an environment characterised by inconsistent or chaotic family life due to substance misuse or parental mental illness (‘Household Dysfunction’). Despite the cogence of this solution, only the internal consistency of ‘Direct Experience of Abuse’ surpassed the reliability threshold of *α* = .70. However, due to the preliminary nature of the current study, the three factors were used in subsequent analyses.

### Placement History

Although gender had no significant bearing on the likelihood of experiencing any of the TALE constructs, one striking finding which emerged from the correlational analyses of TALE scores with background variables was the small but significant correlation between age at admission to care and ‘Direct Experience of Abuse’, and the inverse relationship between age at admission and ‘Household Dysfunction’. Although effect sizes were small, it is possible to hypothesise that children from dysfunctional households which are known to social services may be taken into care at a younger age (before experiencing abuse) because of known parental difficulties, such as substance misuse and mental illness, before the child’s birth (Broadhurst et al., 2018). By contrast, children whose families do not have existing contact with social services may enter care at a later age when child protection issues are raised as the result of evidence of direct harm to the child. Although this explanation is speculative, it may account for the age trend within these two TALE factors.

Related to this finding is the observed relationship between being placed with a sibling and less exposure to traumatic events, compared to children placed without a sibling. Once again, this could be tentatively explained for several reasons: children may be separated from their siblings due to a high level of inter-sibling aggression stemming from traumatic experiences; children with several siblings may ‘share’ the burden of traumatic experiences, each being exposed to fewer; or perhaps solo children are simply more likely to experience neglectful and abusive caregiving. This aligns with emerging evidence that the *impact* of traumatic events is significantly less detrimental to the wellbeing of looked-after children who continue to live with a sibling (Meakings et al., 2017). A sibling relationship may provide a ‘buffering’ effect of social support for managing and processing experiences of adversity (Perricone et al., 2014). Although this preliminary finding does not constitute strong evidence about the reasons why children with greater trauma and adversity may be placed without a sibling, it suggests that a child’s background should be considered by social workers contemplating separating siblings into different placements.

### Trauma and Psychological Wellbeing

There is now substantial evidence that exposure to trauma in childhood can have significant effects on child development and subsequent outcomes. Although the neurobiological mechanisms by which traumatic experiences become internalised and embodied are still under investigation (Bucci et al., 2016; van der Kolk, 2005), robust associations have been consistently found between the experience of neglect and abuse and poor emotional and behavioural wellbeing in childhood (Cheong et al., 2017; Hughes et al., 2017). The moderate positive correlation between the overall TALE score and total difficulties score on the SDQ reported here is therefore congruent with existing research. Moreover, the overall TALE score was also correlated with the SDQ’s likelihood of diagnosis rating. Taken together, these findings provide some evidence of the TALE’s predictive validity. However, this present study is unable to report the concurrent validity of the TALE. A future study would be required to explore whether scores on the TALE converge with other ACEs measures, but this was beyond the scope of a study which used this measure as part of an active clinical assessment process. Likewise, it will be necessary to establish test–retest reliability, but it was deemed unreasonable to request that social workers complete the same measure more than once with no expected change in result.

One way in which the TALE is distinct from other comparable measures is in the inclusion of an Impact section to demonstrate the effect which the respondent believes each event has had on the child’s wellbeing and presentation. This scale demonstrated strong convergent validity with the impact of difficulties score from the SDQ (*r*_*s*_ = .33). The strength of this correlation indicates that social workers identified that current presentations, confirmed by the carer-report SDQ, were rooted in the perceived impact of past experiences. In this way, the impact of traumatic and adverse experiences can be viewed as a foundation for subsequent challenging behaviours, which in turn have a negative impact on children’s social, educational, and emotional functioning. As the first ACEs measure which includes a distinct impact scale, the TALE recognises the importance of exploring not only the *number* of adverse experiences a child has had, but also their subsequent effect on the child. This is important in the context of research about resilience: although there is an association between exposure to adversity and disrupted child development, some children do not experience negative developmental consequences after experiencing ACEs (Bellis et al., 2018). Measures which screen for exposure to ACEs must therefore also provide insight into the impact which these experiences have had on the child.

Exploring the subscales of the SDQ in relation to the TALE factors also yielded interesting relationships. It is perhaps unsurprising that ‘Direct Experience of Abuse’ was significantly correlated with each of the SDQ subscales except conduct problems, since the experience of abuse has previously been connected to developmental psychopathology across a breadth of social and emotional domains (Tarren-Sweeney, 2008). However, one other interesting relationship emerged: ‘Witnessing Harm’ and ‘Household Dysfunction’ were both significantly associated with conduct problems on the SDQ, whereas ‘Direct Experience of Abuse’ was not. This finding could be partly understood from a behavioural perspective: children who grow up in chaotic and unpredictable environments, characteristic of neglectful ‘Household Dysfunction’, may struggle to learn consistent behavioural models from interactions with inconsistent adults (Jaffee et al., 2012; Mensah & Kiernan, 2010). This, in turn, could yield behavioural problems later in childhood.

By contrast, children who are direct targets of abuse may be more likely to enter a withdrawn ‘survival’ mode and seek to avoid capturing their abuser’s attention (Perry & Sullivan, 2014). Thus, emotional difficulties may be internalised rather than expressed behaviourally, and the child’s hypervigilance to their dangerous surroundings may develop into attentional difficulties (Lyons-Ruth & Jacobvitz, 2016). It is indeed possible that the correlation between TALE Exposure score and number of previous placements is mediated by the extent of behavioural difficulties which the child has presented with (Paine et al., 2021). Although these explanations should be thought of as tentative hypotheses, they concur with contemporary trauma theory which understands behavioural responses to complex trauma as being adaptive to the adverse environments from which they emerge (Simkiss, 2019).

Moreover, the finding that dissociative difficulties were significantly correlated with ‘Direct Experience of Abuse’ on the TALE re-affirms that dissociation is a mechanism which is employed when fundamental survival of the child is under threat (Putnam et al., 1993). Recent research, for example, has shown that dissociative coping strategies are especially prominent in children who have experienced sexual abuse (Martin et al., 2022), and are less likely to be present when a child has not experienced a direct threat to their safety. In this sense, the TALE results reflect the a priori expectation that dissociation is present especially in those children with the most significantly abusive backgrounds, again indicating the TALE’s predictive validity (Lynn & Rhue, 1994). This has clinical implications, as clinicians may be particularly attentive to the possibility of dissociative difficulties in children whose TALE indicates that they have experienced abuse directly.

### Strengths and Limitations

This paper is the first study to report on the psychometric properties of the TALE, drawing on a large sample of looked-after children. The results have successfully demonstrated that the TALE is an overall reliable tool, which has good internal and external validity. However, as a preliminary study, the present paper has a number of limitations which require consideration when using the TALE for clinical practice and research. Firstly, the factor structure of the TALE requires further interrogation. Although the overall reliability of the Exposure scale was acceptable, the factors which the exploratory factor analysis yielded lacked reliability. It is therefore crucial that findings related to these constructs are treated cautiously. However, the subscales did discriminate between scores on SDQ subscales and showed acceptably low levels of inter-correlation.

Furthermore, the TALE currently does not have an indicative clinical cut-off score. As most previous ACEs scales have shown a dose–response effect (e.g., Felitti et al., 1998), it may not be possible to detect one point of the Exposure scale at which clinical difficulties are particularly likely. Conventionally, an ACE score of five or more has been taken to indicate likely negative impacts on future health (Bellis et al., 2017), but this threshold requires statistical confirmation. It is also possible that the TALE does not capture all of the possible adverse or traumatic experiences which a child in care *may* have experienced. For instance, omission of items relating to exposure to war may improve the brevity of the measure, but this may be a significantly impactful experience in the life of an unaccompanied asylum-seeking child in care. To ensure content validity, it will be important to ensure that all aspects of trauma and adversity in the looked-after children population are captured by the items on the TALE. Additionally, there was a lack of high-quality case record data from other sources, such as social work records, which could demonstrate convergent validity with the TALE. As a retrospective tool to assess past experiences, it will be important to confirm that the TALE accords with other available records of children’s pre-placement experiences.

Lastly, the limitations of all ACEs tools equally apply to the TALE: cumulative measures of adversity and trauma are limited in their ability to calculate risk of mental and physical health difficulties on a case-by-case basis (Danese, 2019). However, the purpose of the TALE is to pragmatically capture an impression of the known information about the child’s past to contribute to clinical formulation and the planning of psychological interventions. In this purpose, the TALE has been demonstrated to be a practical and valid tool.

## Directions for Future Research

Future research could further explore the factor structure of the TALE by confirmatory factor analysis. Items on the TALE may need refining based on the factor structure detected in the present study, and the internal consistency of each factor will need to meet the threshold value to be considered acceptable. Further research may also explore the properties of the Impact scale in greater depth. Additionally, gathering data from a normative sample of children would enable the estimation of a benchmark score, against which children with remarkably high levels of adversity could be distinguished. It would be valuable for future research to explore clinician’s perspectives about integrating the information from the TALE into a wider assessment process. Moreover, research may address how past experiences recorded by the TALE relate to the extent of security and emotional wellbeing achieved during the course of a placement. Finally, still little is known about the specific pathways between individual ACEs and mental health outcomes in childhood. The exploratory relationships between types of adversity and subsequent psychosocial outcomes presented here provide a foundation for further longitudinal research in this area.

## Conclusion

The present study provides evidence that the Trauma and Adverse Life Events screening tool (TALE) is a valid tool for assessing exposure to, and the impact of, the adverse childhood experiences of looked-after children. A high prevalence of adversity and trauma were detected by the TALE in this population, and associations between traumatic experiences and subsequent behavioural and emotional difficulties were reported. These findings strongly support the approach of trauma-informed care. It is important to contextualise looked-after children’s presentations by gathering information about their past experiences, and the TALE is proposed as an effective tool for this in clinical practice.

## Data Availability

The participants of this study did not give written consent for their data to be shared publicly, so supporting data is not available.

## Appendix

TALE Factors with Descriptive Statistics

**Table:**

TALE Factor	Items	Mean (*SD*)	Skewness	Kurtosis	Cronbach’s *α*
1) Direct Experience of Abuse	1, 3, 4, 7, 8, 9, 10	13.92 (*3.00*)	-.13	-.38	*α* = .76
2) Witnessing Harm	1, 5, 6, 13, 14	8.68 (*2.31*)	.17	-.83	*α* = .57
3) Household Dysfunction	11, 12, 13	6.43 (*1.84*)	-.43	-.76	*α* = .58
